# Reduced muscle activity variability in lumbar extensor muscles during sustained sitting in individuals with chronic low back pain

**DOI:** 10.1371/journal.pone.0213778

**Published:** 2019-03-14

**Authors:** Inge Ringheim, Aage Indahl, Karin Roeleveld

**Affiliations:** 1 Department of Neuromedicine and Movement Science, Faculty of Medicine and Health Sciences, NTNU–Norwegian University of Science and Technology, Trondheim, Norway; 2 Clinic Physical Medicine and Rehabilitation, Vestfold Hospital Trust, Stavern, Norway; Hochschule Trier, GERMANY

## Abstract

The purpose of this study was to investigate muscle activity variability within and between the right and left side of lumbar muscles in patients with chronic low back pain (cLBP) compared to healthy controls (HCs) during sustained quiet sitting. Surface electromyographic (EMG) signals were collected bilaterally from the lumbar muscles with 2 high density surface EMG grids of 9x14 electrodes. Root mean square values (RMS) over 1-sec epochs of all bipolar EMG leadings were obtained. Between-sides alternating activation was computed, as well as temporal- and spatial variability within the electrode grids through the coefficient of variation and correlations between RMS distributions. The subjective influence of sitting was evaluated by the rating of perceived exertion and the amount of LBP on a numeric pain rating scale. Compared to HCs, the patients with cLBP had lower temporal (*p* = 0.03) and similar spatial muscle activity variability during sitting, despite a more variable sitting position. This did not result in increased muscle fatigue indicated by EMG, but the patients with cLBP reported higher levels of RPE during- and more LBP after the sitting and as a consequence ended the sitting earlier than HCs (*p* < 0.01). Present findings lend support to the presence of less tolerance for low-level static muscle load in patients with cLBP.

## Introduction

Low back pain (LBP) is a common health complaint with a global lifetime prevalence of about 40% [1]. Specific pathological causes of LBP like infection, tumor, osteoporosis, fracture or structural deformity are rare, generally reported as less than 15% [2, 3]. This leaves the majority of LBP labelled as non-specific [1, 4]. Therefore, rather than structural derangement, a functional disturbance in the complex system that coordinates the network of paraspinal muscles could be the background for non-specific LBP [5]. Moreover, pain itself may induce guarded behavior and fear which in turn may induce altered muscle activation and monotonous movement patterns [6–8], contributing to a transition from acute to chronic LBP [1]. Chronic LBP (cLBP) is defined as non-specific LBP lasting longer than 12 weeks [2]. A review from 2012 using 86 estimates, reported a global prevalence of cLBP of on average 20% with a standard deviation of 10% [9]. Moreover, the European guidelines for the management of chronic nonspecific low back pain [1] conclude that 12% of the general population reported to be disabled by cLBP.

During low force activities, reduced trunk motor variability is associated with increased muscle fatigue, decreased endurance and increased pain and may therefore play a role in the cause of cLBP [10, 11]. Altered neuromuscular function in patients with cLBP has been shown during different tasks [11–15]. However, the observed muscle activation pattern in patients with cLBP is highly variable and inconsistent [16]. One reason for this inconsistency could be that most studies investigating LBP utilized classic bipolar surface electromyography (EMG) where one signal detected by electrodes usually covering about 1 cm by 3 cm of the skin above the muscle is analyzed.

However, in the lumbar region nearly 70 muscles of variable size are contributing to several possible actions and hence exert various forces and actions on the spinal motion segments [17]. In this way, the numerous back muscles provide a pool of possible “motor solutions” that may be recruited to suit the needs of the vertebral column. In contrast to classic bipolar EMG, high-density surface electromyography (HDsEMG) applies multiple (from about 30 to over 100) small electrodes in a grid over the skin above the muscle of investigation. HDsEMG can therefore reveal information from bigger portions of lumbar muscles and information of the spatial distribution of the activation [18, 19]. The change of the average EMG amplitude during sustained sitting can provide a representative and therefore possibly more stable representation of the activation changes of the whole muscle group [11, 20]. Previously, we have shown indications for relations between frequency of alternating activation between the left and right sides of the lumbar muscles, spatial and temporal variation in muscle activation at both sides and manifestations of muscle fatigue development during sustained sitting in healthy subjects [20]. Moreover, Falla et al [11] showed a significant shift in lumbar muscle activation during dynamic tasks in healthy controls, but not in cLBP. Whether or not persons with cLBP also have altered side-to-side alternating activation and temporal and spatial variation during sustained sitting is unknown.

Therefore, the purpose of the present study was to investigate temporal and spatial muscle activation variability within, and alternating activation between the right and left sides of lumbar muscles in patients with cLBP compared to healthy control persons with HDsEMG during sustained quiet sitting. Systematic temporal changes during sitting were evaluated by linear regression of the EMG amplitude and frequency content and temporal variation by the coefficient of variation around the de-trended EMG amplitude. Spatial variability in the EMG amplitude was explored through the average spatial coefficient of variation and correlations between the amplitude distribution at different time instances. We hypothesized that patients with cLBP would have less variable muscle activation and would be more affected by the sustained sitting showing in more signs of muscle fatigue, reduced sitting time and increased pain.

## Methods

### Subjects

Eighteen patients (13 males and 5 females) with cLBP and 32 healthy controls (HCs; 16 males and 16 females) in the age range 29 to 53 years were included in the study (Table 1). The patients with cLBP were recruited from the outpatient clinic at Vestfold Hospital Trust, were diagnosed with non-specific low back pain of at least 3 months duration and were referred from general practitioners. The HC were without back pain in the previous year or back pain lasting longer than one week in the previous 3 years. Exclusion criteria were anamnesis of medical or drug abuse, surgery on the musculoskeletal system of the trunk, known congenital malformation of the spine or scoliosis, systemic-neurological-degenerative disease, history of stroke, psychiatric disorder and pregnancy. Moreover, due to test protocol risks, persons with abnormal blood pressure were also excluded from the study. Participants were asked not to perform any back-straining exercises 48h prior to examination and not to use any medications except for Paracetamol or Ibuprofen preparations during one week before participation. Information of lumbar muscle fatigue, variation in muscle activation and gender differences during sustained sitting from the 32 HCs has previously been published [20].

**Table 1:** Subject characteristics.

*Characteristic*	*HC (n = 25)*	*cLBP (n = 18)*	*t or U (p)*
	*Mean (SD)*	*Mean (SD)*	
*Age (year)*	*39*.*9 (6*.*6)*	*40*.*8 (7*.*8)*	*-0*.*45* ^*t*^ *(0*.*66)*
*Height (cm)*	*174*.*1 (9*.*6)*	*177*.*6 (7*.*9)*	*-1*.*27* ^*t*^ *(0*.*21)*
*Weight (kg)*	*70*.*0 (12*.*1)*	*73*.*3 (9*.*6)*	*179*.*5* ^*U*^ *(0*.*26)*
*BMI (kg/m*^*2*^*)*	*22*.*9 (2*.*2)*	*23*.*2 (2*.*1)*	*-0*.*39* ^*t*^ *(0*.*70)*
*Muscle depth (mm)*	*9*.*6 (2*.*4)*	*10*.*2 (3*.*6)*	*-0*.*58* ^*t*^ *(0*.*57)*
*PAL (0–10)*	*7*.*2 (1*.*9)*	*7*.*4 (1*.*5)*	*210* ^*U*^ *(0*.*71)*
*Average pain last week (0–10)*		*6 (2*.*6)*	
*ODI*		*26*.*9 (9*.*6)*	
*TSK (13–52)*		*27*.*1 (7*.*4)*	

Mean and standard deviation (SD) of the low back pain patients (cLBP) and healthy control subjects (HC) characteristics.

Abbreviations: BMI; Body Mass Index, ODI; Oswestry Disability Index, PAL; Physical Activity Level, TSK; Tampa Scale of Kinesiophobia.

Group differences evaluated with ^*t*^ independent T-test or ^*U*^ Mann-Whitney U test with the level of significance (p).

Due to poor signal quality from muscles covered with subcutaneous soft tissue and fascia > 15 mm, seven HCs were excluded and 25 HCs were included in final analyses (13 males and 12 females).

The project was approved by the Regional Committee for Medical Research Ethics (REK) in the South-Eastern Norwegian Regional Health Authority (S-08630a, 2008/1585) and all subjects signed an informed consent prior to participation.

### Experimental setup and procedure

The same experimental procedure was used as in [20], and for details one is referred to this paper. In short, the participants’ characteristics were obtained through a custom-made questionnaire. Ultrasound measurements 3 cm lateral of the spinous process at the L3-L4 level were used to determine the subcutaneous soft tissue and fascia thickness.

**Fig 1:**
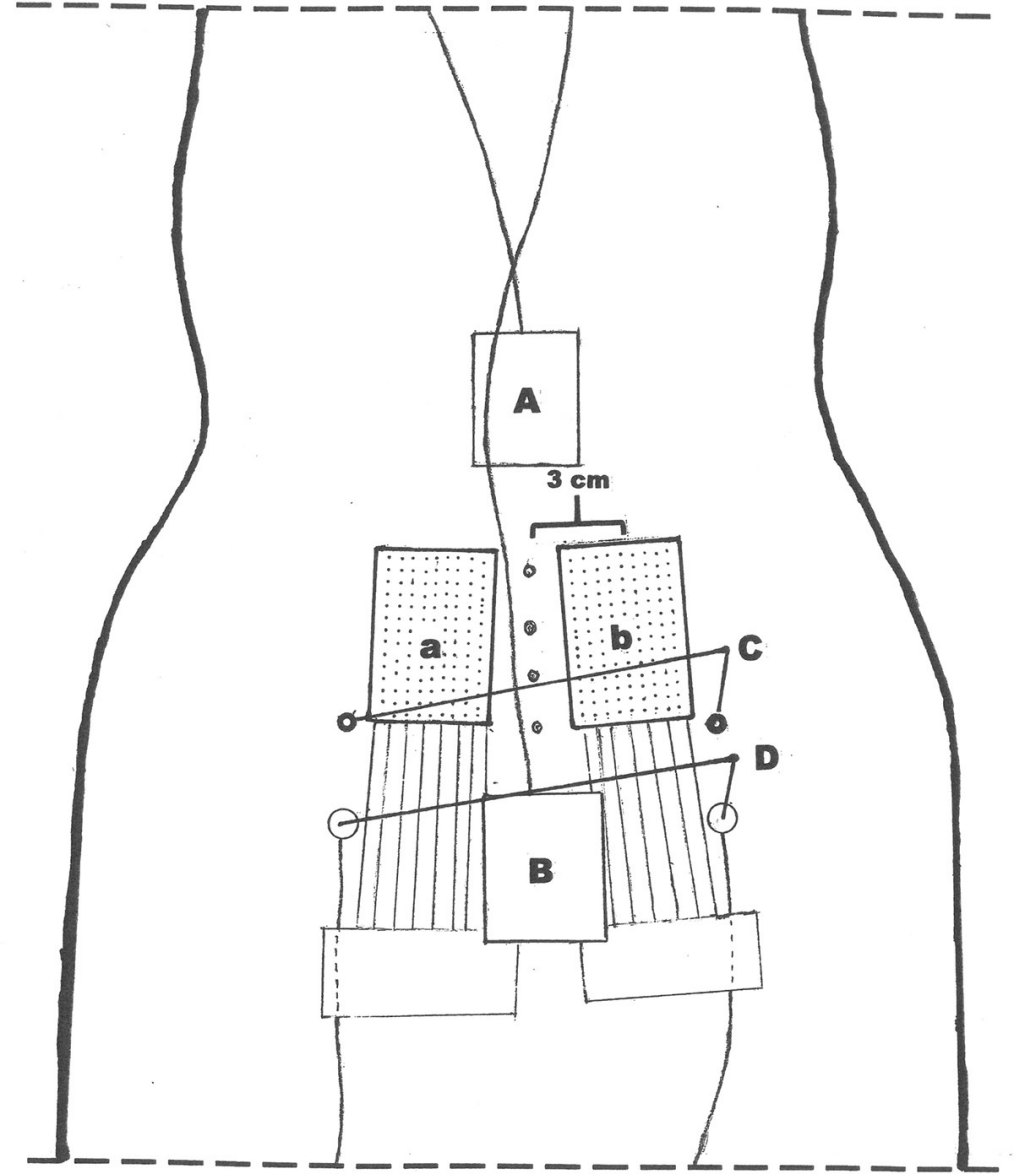
Placement of inclinometers and HDsEMG grids. The drawing demonstrates the electrode grids placement a) and b), center of the grid located 3 cm from the spine process and with the base of the grid at the level of the posterior superior iliac spine C). A) and B) demonstrates the position of the trunk and pelvis inclinometers, one in the lower part of the thoracic spine, and one on the sacrum.

The sitting position was controlled by two inclinometers placed on the back at the Th.12 and S1-level (Fig 1). The signals were collected with a sampling frequency of 1500 Hz in MyoResearch XP Master Edition (Noraxon). A target position from the inclinometer at Th 12 was given on a computer screen.

Two HDsEMG grids consisting of 126 (9 medial-lateral columns by 14 caudal-cranial rows) gelled Ag-AgCl electrodes with 4 mm inter electrode distance were attached to the skin with double-sided tape. Prior to electrode placement, the skin was prepared with an abrasive paste and an electrode gel was applied on the electrodes. The center of the electrode grids was placed 3 cm from the spine process with the base of the grids at the level of the posterior superior iliac spine. Two 128-channel ActiveTwo amplifier systems (BioSemi, Amsterdam, The Netherlands), were used for data collection with a sample rate of 2048 Hz per channel (24 bit, 4th order Delta-Sigma modulator with 64x oversampling; fixed first order analog anti-aliasing filter, -3dB at 3.6 kHz), using data acquisition software (MyoDaq) developed at the Department of Clinical Neurophysiology of the Radboud University Nijmegen Medical Center.

In a seated position, the subjects performed three 5s maximal contractions of back extension against resistance of a strap around the upper part of the trunk. Three minutes rest was taken between the 3 trials. After another break of 10 minutes, the participants were asked to maintain a trunk inclination of 5° forward from vertical for 30 minutes or until “task failure”. Task failure was defined as a deviation from the target inclination of 1 degree for more than 3 s. Every five minutes, subjects rated their perceived exertion (RPE) experienced during the sustained sitting on a scale ranging from 6–20 (Borg, 1982).

### Data analyses

First, HDsEMG channels with poor quality were removed. Then, the signals were band pass filtered (30 – 300Hz) and a bipolar spatial filter was applied in the cranial-caudal direction with inter electrode distances of 12 mm, leaving 9 columns and 11 rows with bipolar EMG. For each bipolar signal, the root mean square (RMS) and median frequency (MDF) were calculated in 1s non-overlapping epochs. The RMS values were normalized to the 1s highest RMS value obtain during the maximal contractions. It is important to note that the participants with cLBP reached lower levels of RMS during the MVCs, indicating a possible motor unit recruitment failure, hampering comparison of normalized RMS data with HC. Therefore, absolute RMS data are also presented. For each grid and all epochs, the overall grid average was obtained.

The slope of a linear regression of the changes in grid averaged RMS and MDF values during sustained sitting were obtained (RMSslope and MDFslope) to quantify systematic changes over time.

The frequency of the alternating activation between left and right lumbar muscles was used to quantify the variability in muscle activity between sides as previously described in detail [20]. In short, the detrended grid average RMS signal was normalized to the second highest RMS value from each signal. The second highest value was used due to the possible effects of transient artifacts in the signals on the peak RMS value. The number of periods with a difference between the two sides exceeding 30% was counted and divided by time, resulting in a frequency.

The temporal variations in amplitude were quantified as the coefficient of variation of the de-trended grid averaged signals (COV_TEMPORAL_ = 100*SD de-trended RMS /mean RMS).

To explore the spatial variability of the EMG amplitudes within the electrode grid during the quiet sitting, the coefficient of variation of all RMS signals in the grid per epoch was obtained and averaged over the sitting period (COV_SPATIAL_).

The RMS distribution changes over time were quantified by the correlation coefficients between RMS values of different epochs. Correlation coefficients (CCT) for all possible combinations were obtained during the sustained sitting task resulting in a matrix of correlations. The median value (CCT_MED_) was taken where low CCT_MED_ thus indicates a large variation in RMS distribution.

None of the calculated EMG variables differed significantly between the electrode-grids placed on the left and right side of the back. Therefore, all EMG variables were averaged bilaterally.

The average medio/lateral and anterior/posterior position for each second was computed. From the inclinometer data, the absolute change and variability (SD) in position during sitting was calculated.

### Statistical analyses

The statistical analyses were performed with the software PASW Statistics 21. A Shapiro-Wilk W-test for normality was performed on all dependent variables before statistical analysis. As almost all variables turned out to have a non-normal distribution, non-parametric statistics were applied. For within subject changes the Wilcoxon signed rank test was performed and the Mann-Whitney U test was applied for differences between groups. The slopes for MDF and RMS were tested against zero with the one sample Wilcoxon test. The significance level for all tests was set to *p* < 0.05, and comparisons were performed two tailed. The non-parametric testing hampered multivariate testing and no correction for multiple comparisons was undertaken, impeding a more exploratory nature of the study.

## Results

The BMI and the depth of the lumbar muscles measured with US (skin plus subcutaneous fat layer) were, together with other subjects characteristics, similar in patients with cLBP and HCs (all *p*-values > 0.1) (Table 1). Eight patients with cLBP (~40%) and one HC ended the sitting task before the scheduled 30 minutes due to experienced perceived exertion, pain and discomfort in the sitting position. On average, the LBP patients had significant shorter sitting time (median (IQR) HC; 30 (0), cLBP; 20 (23), (*p* ≤ 0.01).

During the sitting, only very small changes in position were observed (all changes for trunk and pelvis position < 2.1°). However, the change in medial-lateral direction of the trunk position was significant larger in patients with cLBP compared to HCs (*p* < 0.01). Moreover, during the sitting the patients with cLBP had increased variation in the trunk and pelvis position compared to the HCs (Table 2). This was statistically significant (*p*-values ≤ 0.05) in all directions except for the anterior-posterior direction of the pelvis (*p* = 0.25). Moreover, the patients with cLBP reported higher RPE at the start and after the sitting and had a greater change in the RPE after the sitting (all *p*-values ≤ 0.02) (Table 3).

**Table 2:** Muscle activation during sitting.

	*HC (n = 25)*	*cLBP (n = 18)*	*U (p)*
	*Median (IQR)*	*Median (IQR)*	
*Start EMG RMS (uV)*	*481 (391; 919)*	*265 (173; 487)*	*106 (<0*.*01)*
*Start EMG RMS (%RMSmvc)*	*19*.*7 (12*.*6; 25*.*2)*	*24*.*2 (18*.*1; 43*.*9)*	*145 (0*.*05)*
*Start EMG MDF (Hz)*	*101*.*8 (96*.*7; 113*.*9)*	*125*.*9 (117*.*9;148*.*4)*	*80 (< 0*.*01)*
*EMG RMS slope (uV/min)*	*3*.*9 (0*.*9; 10*.1)**	*3*.*5 (0*.*2; 9*.2)**	*201 (0*.*56)*
*EMG RMS slope (% RMSmvc/min)*	*0*.*13 (0*.*03; 0*.28)**	*0*.*27 (0*.*05; 0*.51)**	*281 (0*.*17)*
*EMG MDF slope (Hz/min)*	*-0*.*02 (-0*.*19; 0*.*19)*	*-0*.*06 (-0*.*29; 0*.*07)*	*189 (0*.*38)*

Median and interquartile range (IQR) of the low back pain patients (cLBP) and healthy control subjects (HC) of the grid-averaged root mean square amplitude (RMS) and median frequency (MDF) of EMG collected from the lumbar muscles at the start of the sitting task and the slope of the change of these variables during sustained sitting. RMS values are presented both in uV and as a percentage of the maximal RMS obtained under a maximal voluntary contraction (%RMSmvc). U-values and significant levels (p) of the group effect resulting from the Mann-Whitney U test are also included.

** significant different from zero.

**Table 3:** Variation in posture and muscle activation during sitting.

	*HC*	*cLBP*	*U (p)*
	*Median (IQR)*	*Median (IQR)*	
*trunk a/p SD (°)*	*0*.*16 (0*.*11; 0*.*24)*	*0*.*21 (0*.*19;0*.*30)*	*315 (0*.*03)* *
*trunk m/l SD (°)*	*0*.*41 (0*.*30; 0*.*76)*	*0*.*73 (0*.*66;1*.*29)*	*348 (<0*.01)*
*pelvis a/p SD (°)*	*0*.*74 (0*.*35; 1*.*52)*	*0*.*81 (0*.*55; 1*.*99)*	*270 (0*.*27)*
*pelvis m/l SD (°)*	*0*.*26 (0*.*20; 0*.*51)*	*0*.*47 (0*.*27; 1*.*41)*	*310 (0*.04)*
*EMG alternating frequency (min*^*-1*^*)*	*8*.*0 (4*.*8; 9*.*1)*	*7*.*6 (4*.*3; 11*.*7)*	*201 (0*.*56)*
*EMG COV*_*TEMPORAL*_ *(%)*	*8*.*7 (7*.*4; 10*.*9)*	*7*.*0 (3*.*0; 9*.*4)*	*135 (0*.03)*
*EMG COV*_*SPATIAL*_ *(%)*	*26*.*1 (19*.*1; 34*.*8)*	*27*.*9 (16*.*1; 44*.*1)*	*255 (0*.*46)*
*EMG CCT*_*MED*_ *(r)*	*0*.*89 (0*.*83; 0*.*93)*	*0*.*93 (0*.*71; 0*.*97)*	*202 (0*.*57)*

Median and interquartile range (IQR) of the low back pain patients (cLBP) and healthy control subjects (HC) of the variables showing variation in posture and muscle activation during the sustained sitting task. Variation in posture: Coefficient of variation (COV) of the position in anterior-posterior (a/p) and medial-lateral (m/l) direction for the trunk and pelvis. Variation in muscle activation investigated by root mean square amplitude (RMS) of EMG collected from the lumbar muscles obtained with bipolar leadings. Frequency of alternating activation between the left and right side of the back muscles, the coefficient of temporal variation of the grid-average RMS (COV_TEMPORAL_), the average coefficient of spatial variation of the RMS within the electrode grid (COV_SPATIAL_) and the RMS distribution change (CCT_MED_). U-values and significant levels (p) of the group effect resulting from the Mann-Whitney U test are also included.

* significant group difference.

### Muscle activation and variability during sustained sitting

**Fig 2:**
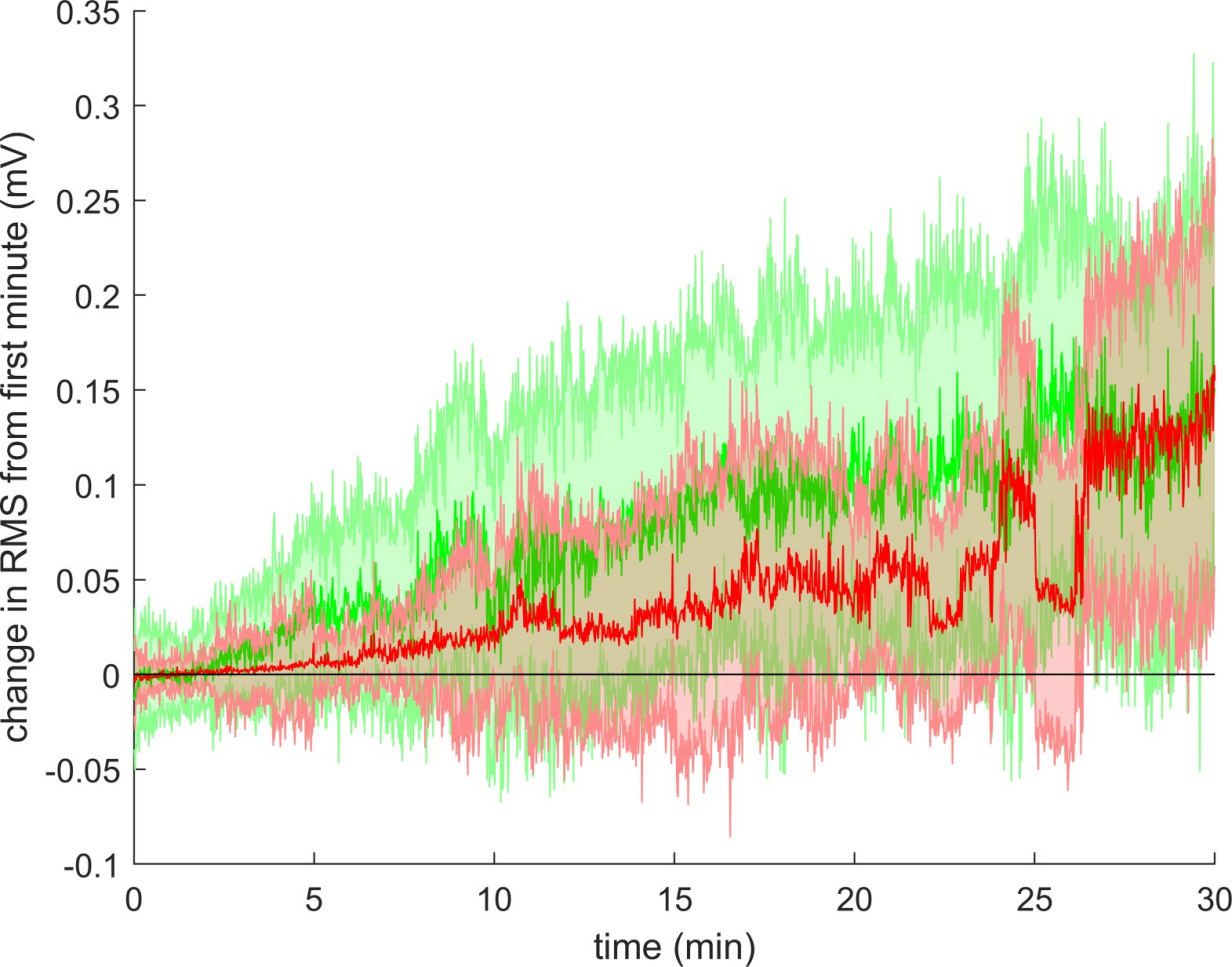
Change in RMS during sitting. The figure shows the change in average RMS during sitting in cLBP patients (red) and healthy controls (green). Solid line: group median. Shaded area: inter quartile range. Since not all participants could sit for 30 minutes, the number of participants presented in this figure are less at the end than at the beginning.

The absolute RMS amplitude at the start of the sitting was lower in the patients with cLBP compared to HCs (*p* < 0.01), while this value normalized to the RMS obtained during MVC had a tendency to be higher in patients with cLBP (p = 0.06) (Table 2). Moreover, the MDF at the start of the sitting was higher in patients with cLBP (*p* < 0.01). During the sitting, both the absolute and relative RMS increased significantly in both groups (both *p* < 0.01; Table 2 and Fig 2) while the MDF remained unchanged.

There were no group differences in the change of absolute RMS (*p* = 0.56), relative RMS (*p* = 0.17) or MDF (*p* = 0.38) during the sitting (Table 2).

Table 3 summarizes the EMG variability of lumbar muscles. The patients with cLBP had significantly lower (*p* = 0.03) temporal variation (COV_TEMPORAL_) in grid-averaged RMS. The alternating frequency was not different between patients with cLBP and in HCs, (*p =* 0.56). The spatial variability of the EMG amplitudes within the electrode grids during the sitting (COV_SPATIAL_) and the EMG spatio-temporal correlation (CCT_MED_) were high without group differences (COV_SPATIAL_; *p* = 0.46, CCT_MED_; *p* = 0.56).

### Muscle activation during maximal voluntary contractions before and after sustained sitting

Results from muscle activation during MVC before and after the sitting are shown in Table 4. The patients with cLBP had lower maximal RMS during MVC before and after the sitting (*p* ≤ 0.05). Both groups had reduced RMS_MVC_ after the sitting, significant for the patients with cLBP (*p* = 0.04). However, the change in RMS_MVC_ was not significant different between the 2 groups (*p* = 0.30). The patients with cLBP had higher pain ratings in the beginning and at the end of the sitting (both *p* < 0.01), and the patients increase in LBP was significant (*p* = 0.01).

**Table 4:** Differences from before to after sustained sitting.

*HC n = 25*				*cLBP n = 18*			*U (p)*
	*Pre SS*	*Post SS*	*Z(p)*	*Pre SS*	*Post SS*	*Z (p)*	
*RMS*_*MVC*_ *(mV)*	*3*.*1 (1*.*8–5*.*3)*	*2*.*5 (2*.*0–5*.*3)*	*-0*.*5 (0*.*6)*	*1*.*3 (0*.*6–2*.*8)*	*1*.*2 (0*.*6–2*.*1)*	*-2*.*1 (0*.*04)*	*183 (0*.*30)*
*NPRS (0–10)*	*0*.*0 (0*.*0–0*.*0)*	*0*.*0 (0*.*0–0*.*0)*	*-1*.*3 (0*.*2)*	*3*.*5 (1*.*8–6*.*0)*	*6*.*0 (3*.*5–8*.*0)*	*-2*.*6 (0*.*01)*	*370 (<0*.*01)*
*Perceived exertion*	*6*.*0 (6*.*0–8*.*5)*	*13*.*0 (11*.*0–15*.*0)*	*-4*.*3 (<0*.*01)*	*9*.*0 (6*.*0–13*.*0)*	*19*.*0 (17*.*0–19*.*3)*	*-3*.*7 (<0*.*01)*	*336 (0*.*01)*

Median and interquartile range (IQR) of pre and post sustained sitting (SS) results in low back pain patients (cLBP) and healthy controls (HC) of muscle activation during maximal voluntary contraction in sitting trunk extension (RMS_MVC_), rating of low back pain (NPRS) and rating of perceived exertion. Results from the Wilcoxon signed rank test (within subjects change) Z (p) and Mann-Whitney U test (group differences in change) U (p) are included.

## Discussion

The aim of the present study was to investigate lumbar muscle activation during, and as a result of, sustained quiet sitting in patients with cLBP compared to healthy controls (HCs).

The patients with cLBP had on average a significantly shorter sitting time than HCs. Moreover, they developed a significant increase in pain and showed a larger increase in perceived exertion compared to HCs during the sitting. Our results support therefore the presence of less tolerance for low-level static muscle load in patients with cLBP induced by the quiet sitting. This is in agreement with other studies [21, 22], and our observations during prolonged standing [23].

As instructed, during this quiet sitting task, both the HCs and the patients with cLBP had very little variation in their posture. Nevertheless, this postural variation during sitting was significantly larger in patients with cLBP compared to the HCs. Perceived discomfort from the quiet sitting may well be compensated by tiny changes in lumbar intervertebral positions, and hence increase variation in lumbar muscle system. This is also in agreement with our previous observations during prolonged (not quiet) standing, where patients with cLBP changed standing posture more frequent than HCs [21]. The perception of muscle fatigue, musculoskeletal pain and discomfort in the postural control system are believed to initiate such changes in posture [24] and it is not likely that this increased variation would cause the discomfort and pain.

The initial activation level in our study was on average about 20% RMS_MVC_, and somewhat higher in patients with cLBP. A significant increase in RMS and RPE was observed in both patients with cLBP and HCs indicating an on-going fatiguing process during sitting. The target sitting position (5° trunk inclination) in our experimental setup probably led to a higher activation level in lumbar extensor muscles during the sitting than what is usually observed (< 10%RMSmax) [25].

Compared to HCs, the patients with cLBP seems to have reduced temporal variability in lumbar muscle activation during the quiet sitting. This is in line with observations of reduced motor variability in chronic pain conditions and linked to muscle fatigue [10]. Reduced temporal variability in muscle activity has been shown to induce local muscle fatigue even under isometric muscle contractions at a very low level [26]. Although this reduced temporal variability was accompanied by increased pain and perceived exertion in the patients with cLBP, the myoelectric manifestation of muscle fatigue (increased RMS or decreased MDF) were not different between the two groups.

During sitting, both patients with cLBP and HCs had little variation in the RMS distribution over time (high CCT_MED_; around 0.9) in lumbar muscles (Table 3). Thus, the slightly increased variation in position accompanied by a significant reduction in temporal RMS variation was not accompanied by a significant increase in variation in RMS distribution over time. This contrasts the findings of Falla et al. [11] where a significant shift in lumbar muscle activation during dynamic tasks was seen in healthy controls, but not in cLBP. However, differences in the task makes direct comparisons difficult.

Our finding of little variation of RMS distribution over time although increase of variation of position lend some support to the theory that patients with cLBP may have difficulty to deactivate lumbar muscles despite changing sitting position, and result in constant low level isometric activity resulting in local muscle fatigue and possibly leading to musculoskeletal pain. Reduced ability to relax muscles after activation and shorter rest periods during repetitive tasks have been observed in neck pain patients [27]. However, the lower EMG amplitudes in the patients with cLBP could also indicate reduced activation in the low back muscles. The increased movement variation could be caused by other, non-investigated muscle groups contributing to back extension.

The alternating activation between sides of lumbar muscles was similar in patients with cLBP compared to HCs. Higher frequency of alternating activation in lumbar muscles have previously been linked to increased fatigue development during sustained sitting [20], to fatigue in biceps brachii in healthy persons [28] and pain intensity in trapezius in patients with a chronic musculoskeletal disorder (fibromyalgia) [29]. Similar, spatial variation in muscle activation observed during constant force contractions seem to have positive effect on local signs of muscle fatigue during sustained contractions [30, 31]. The exact mechanism for alternating activation is not clear, although feedback from local muscle fatigue via afferents to α-motoneurones (via interneurons) has been mentioned as a plausible mechanism [32]. The patients with cLBP in our study had similar manifestations of local muscle fatigue in the EMG signal during the sitting (similar increase in RMS) which may explain the observed similar alternating activation. Moreover, the force level of the contraction can be a considerable factor for alternate activity, and the muscle activation level observed in our study may have been too high for true alternating activation to occur. Furthermore, the patients with cLBP in our study had increased postural movement variability, reducing the validity of alternating activation and other muscle activation variability comparisons.

Low maximal RMS during the MVC performed before the sustained sitting, and low EMG and high RPE was observed in patients with cLBP at the start of the sitting (Table 4). This indicates reduced muscle activation, plausibly caused by pain [16]. An interpretation of the increased ratings of perceived exertion at the start of the sitting could be that patients with cLBP already at the start of the sitting were experiencing muscle fatigue.

### Study limitations

Although the number of participants was large enough to detect group differences, the sample size of 18 patients and 25 HC in combination with non-parametric statistics without multi-testing correction opens for a risk of random significant differences. Therefore, especially the results with low statistical significance should be taking with care and seen in view of other studies in the literature.

The HC group were persons without back pain in the previous year or back pain lasting longer than one week in the previous 3 years. Some of those HC participants, therefore probably have had LBP during their life, like most of the healthy general population. Therefore, the results represent differences between persons with chronic LBP and a normal healthy population only and cannot be generalized to a population without any LBP history.

Although we asked the participants not to use any medications except for Paracetamol or Ibuprofen preparations one week before examination and we have the impression that our participants in general are very dutiful, we cannot guarantee that they did not do so. Medications might affect muscle activation.

Even though the electrode grid covers a substantial portion of the lumbar muscles, we acknowledge that fibres of iliocostalis and latissimus dorsalis muscle with substantial extensor action is not well represented by the electrode position in this study.

Low maximal RMS during the MVC performed by the patients with cLBP is of concern. It is an indication of altered voluntary drive, and may be an avoidance behavior due to pain expectancy during muscle contraction. This also hampers the interpretation of group comparisons of normalized RMS values, since the normalized RMS values in patients with cLBP most likely are overestimated. However, conclusions in this paper are mainly based upon temporal and spatial variability of muscle activation, not influenced by the absolute EMG amplitude. Moreover, the change in maximal RMS during MVC performed before and after the sitting was not different in patients with cLBP and HCs, indicating that reduced performance in patients during MVC might not solely be as a result of pain avoidance behavior.

Our study investigated the spatial RMS distribution during sitting in one single position/task, and we recognize that comparing the spatial RMS distribution within participants between tasks with fairly similar postures would increase the validity of these analyses.

### Clinical relevance and future directions

From a clinical view, in contrast to pain-free individuals, patients with cLBP seem to employ different movement strategies. Although guarded movements and stiffened behavior are frequently observed [33, 34], increased variation in sitting position in this study can be seen as a normal response to increased perception of musculoskeletal discomfort and pain. Patients with cLBP seem therefore to have a normal (postural) strategy while sitting, and this strategy probably does not contribute largely to the LBP for patients included in this study. Despite increased variation in sitting position, no increased variability in muscle activation of the low back muscles was observed. Psychological factors are important in LBP [35], and the uncertainty related to what the non-specific LBP represents for the individual may contribute to alterations in activation of trunk muscles, such as relatively higher activation level and reduced variability in muscle activation [10, 11]. Cognitive behavioural interventions seem effective in reducing disability and pain in non-specific LBP [36], and understanding the mechanisms by which psychological factors can affect the development of cLBP are wanted [37]. A commendable direction for future studies is to evaluate the effect of cognitive behavioural interventions on motor control, muscle activation and psychological systems during activities requiring low-level load (e.g. sitting and standing in different ways) in daily life.

### Conclusions

The patients with cLBP in our study had reduced tolerance for sitting, similar spatial- and lower temporal variability of muscle activation in the low back muscles compared to HCs during sitting, despite increased variability in the sitting position. However, this did not result in increased manifestations of muscle fatigue in the EMG, although the patients with cLBP experienced higher levels of perceived exertion and more pain during sitting. The patients with cLBP might have avoided activation changes in the low back region and compensated this somewhat by activation of other muscle groups, but terminated the sitting early due to this strategy.

## References

[1] Airaksinen O, Brox JI, Cedraschi C, Hildebrandt J, Klaber-Moffett J, Kovacs F, et al Chapter 4. European guidelines for the management of chronic nonspecific low back pain. Eur Spine J. 2006;15 Suppl 2(0):S192–300. 10.1007/s00586-006-1072-1 . 16550448 PMC3454542

[2] Koes BW, van Tulder MW, Thomas S. Diagnosis and treatment of low back pain. BMJ. 2006;332(7555):1430–4. 10.1136/bmj.332.7555.1430 . 16777886 PMC1479671

[3] Hartvigsen J, Hancock MJ, Kongsted A, Louw Q, Ferreira ML, Genevay S, et al What low back pain is and why we need to pay attention. Lancet. 2018;391(10137):2356–67. 10.1016/S0140-6736(18)30480-X . 29573870

[4] Maher C, Underwood M, Buchbinder R. Non-specific low back pain. Lancet. 2017;389(10070):736–47. 10.1016/S0140-6736(16)30970-9 . 27745712

[5] Holm S, Indahl A, Solomonow M. Sensorimotor control of the spine. J Electromyogr Kinesiol. 2002;12(3):219–34. . 12086817 10.1016/s1050-6411(02)00028-7

[6] Indahl A, Kaigle A, Reikeras O, Holm S. Electromyographic response of the porcine multifidus musculature after nerve stimulation. Spine (Phila Pa 1976). 1995;20(24):2652–8. . 8747243 10.1097/00007632-199512150-00006

[7] Indahl A, Kaigle AM, Reikeras O, Holm SH. Interaction between the porcine lumbar intervertebral disc, zygapophysial joints, and paraspinal muscles. Spine (Phila Pa 1976). 1997;22(24):2834–40. Epub 1998/02/12. . 9431619 10.1097/00007632-199712150-00006

[8] Indahl A, Kaigle A, Reikeras O, Holm S. Sacroiliac joint involvement in activation of the porcine spinal and gluteal musculature. J Spinal Disord. 1999;12(4):325–30. . 10451049

[9] Hoy D, Bain C, Williams G, March L, Brooks P, Blyth F, et al A systematic review of the global prevalence of low back pain. Arthritis Rheum. 2012;64(6):2028–37. 10.1002/art.34347 . 22231424

[10] Abboud J, Nougarou F, Page I, Cantin V, Massicotte D, Descarreaux M. Trunk motor variability in patients with non-specific chronic low back pain. Eur J Appl Physiol. 2014;114(12):2645–54. 10.1007/s00421-014-2985-8 . 25173095

[11] Falla D, Gizzi L, Tschapek M, Erlenwein J, Petzke F. Reduced task-induced variations in the distribution of activity across back muscle regions in individuals with low back pain. Pain. 2014;155(5):944–53. 10.1016/j.pain.2014.01.027 . 24502841

[12] Geisser ME, Ranavaya M, Haig AJ, Roth RS, Zucker R, Ambroz C, et al A meta-analytic review of surface electromyography among persons with low back pain and normal, healthy controls. J Pain. 2005;6(11):711–26. 10.1016/j.jpain.2005.06.008 . 16275595

[13] Jones SL, Henry SM, Raasch CC, Hitt JR, Bunn JY. Individuals with non-specific low back pain use a trunk stiffening strategy to maintain upright posture. J Electromyogr Kinesiol. 2012;22(1):13–20. 10.1016/j.jelekin.2011.10.006 . 22100719 PMC3246114

[14] Lamoth CJ, Meijer OG, Daffertshofer A, Wuisman PI, Beek PJ. Effects of chronic low back pain on trunk coordination and back muscle activity during walking: changes in motor control. Eur Spine J. 2006;15(1):23–40. 10.1007/s00586-004-0825-y . 15864670 PMC3454567

[15] Freddolini M, Strike S, Lee RY. The role of trunk muscles in sitting balance control in people with low back pain. J Electromyogr Kinesiol. 2014;24(6):947–53. 10.1016/j.jelekin.2014.09.009 . 25287529

[16] van Dieen JH, Selen LP, Cholewicki J. Trunk muscle activation in low-back pain patients, an analysis of the literature. J Electromyogr Kinesiol. 2003;13(4):333–51. . 12832164 10.1016/s1050-6411(03)00041-5

[17] Bogduk N. Clinical anatomy of the lumbar spine and sacrum. 4th ed ed. Edinburgh: Elsevier Churchill Livingstone; 2005. XII, 250 s. p.

[18] Cattarello P, Vinelli S, D'Emanuele S, Gazzoni M, Merletti R. Comparison of chairs based on HDsEMG of back muscles, biomechanical and comfort indices, for violin and viola players: A short-term study. J Electromyogr Kinesiol. 2018;42:92–103. 10.1016/j.jelekin.2018.06.013 . 30015135

[19] Afsharipour B, Petracca F, Gasparini M, Merletti R. Spatial distribution of surface EMG on trapezius and lumbar muscles of violin and cello players in single note playing. J Electromyogr Kinesiol. 2016;31:144–53. 10.1016/j.jelekin.2016.10.003 . 27835831

[20] Ringheim I, Indahl A, Roeleveld K. Alternating activation is related to fatigue in lumbar muscles during sustained sitting. J Electromyogr Kinesiol. 2014;24(3):380–6. 10.1016/j.jelekin.2014.01.011 . 24594079

[21] Nairn BC, Azar NR, Drake JD. Transient pain developers show increased abdominal muscle activity during prolonged sitting. J Electromyogr Kinesiol. 2013;23(6):1421–7. 10.1016/j.jelekin.2013.09.001 . 24135196

[22] Schinkel-Ivy A, Nairn BC, Drake JD. Investigation of trunk muscle co-contraction and its association with low back pain development during prolonged sitting. J Electromyogr Kinesiol. 2013;23(4):778–86. 10.1016/j.jelekin.2013.02.001 . 23489715

[23] Ringheim I, Austein H, Indahl A, Roeleveld K. Postural strategy and trunk muscle activation during prolonged standing in chronic low back pain patients. Gait Posture. 2015;42(4):584–9. 10.1016/j.gaitpost.2015.09.008 . 26404082

[24] Duarte M, Zatsiorsky VM. On the fractal properties of natural human standing. Neurosci Lett. 2000;283(3):173–6. 10.1016/s0304-3940(00)00960-5 . 10754215

[25] Mork PJ, Westgaard RH. Back posture and low back muscle activity in female computer workers: a field study. Clin Biomech (Bristol, Avon). 2009;24(2):169–75. 10.1016/j.clinbiomech.2008.11.001 . 19081657

[26] van Dieen JH, Westebring-van der Putten EP, Kingma I, de Looze MP. Low-level activity of the trunk extensor muscles causes electromyographic manifestations of fatigue in absence of decreased oxygenation. J Electromyogr Kinesiol. 2009;19(3):398–406. Epub 2008/01/08. 10.1016/j.jelekin.2007.11.010 . 18178450

[27] Falla D, Farina D. Neuromuscular adaptation in experimental and clinical neck pain. J Electromyogr Kinesiol. 2008;18(2):255–61. 10.1016/j.jelekin.2006.11.001 . 17196826

[28] Holtermann A, Gronlund C, Ingebrigtsen J, Karlsson JS, Roeleveld K. Duration of differential activations is functionally related to fatigue prevention during low-level contractions. J Electromyogr Kinesiol. 2010;20(2):241–5. 10.1016/j.jelekin.2009.04.011 . 19481957

[29] Holtermann A, Gronlund C, Roeleveld K, Gerdle B. The relation between neuromuscular control and pain intensity in fibromyalgia. JElectromyogrKinesiol. 2011;21(3):519–24. 10.1016/j.jelekin.2011.01.004 21333549

[30] Falla D, Farina D. Non-uniform adaptation of motor unit discharge rates during sustained static contraction of the upper trapezius muscle. Exp Brain Res. 2008;191(3):363–70. 10.1007/s00221-008-1530-6 . 18704381

[31] Farina D, Leclerc F, rendt-Nielsen L, Buttelli O, Madeleine P. The change in spatial distribution of upper trapezius muscle activity is correlated to contraction duration. Journal of Electromyography and Kinesiology. 2008;18(1):16–25. 10.1016/j.jelekin.2006.08.005 17049273

[32] Kouzaki M, Shinohara M. The frequency of alternate muscle activity is associated with the attenuation in muscle fatigue. Journal of Applied Physiology. 2006;101(3):715–20. 10.1152/japplphysiol.01309.2005 16728513

[33] van der Hulst M, Vollenbroek-Hutten MM, Rietman JS, Hermens HJ. Lumbar and abdominal muscle activity during walking in subjects with chronic low back pain: support of the "guarding" hypothesis? J Electromyogr Kinesiol. 2010;20(1):31–8. 10.1016/j.jelekin.2009.03.009 . 19683459

[34] Geisser ME, Haig AJ, Wallbom AS, Wiggert EA. Pain-related fear, lumbar flexion, and dynamic EMG among persons with chronic musculoskeletal low back pain. Clin J Pain. 2004;20(2):61–9. . 14770044 10.1097/00002508-200403000-00001

[35] Chou R, Shekelle P. Will this patient develop persistent disabling low back pain? JAMA. 2010;303(13):1295–302. 10.1001/jama.2010.344 . 20371789

[36] Richmond H, Hall AM, Copsey B, Hansen Z, Williamson E, Hoxey-Thomas N, et al The Effectiveness of Cognitive Behavioural Treatment for Non-Specific Low Back Pain: A Systematic Review and Meta-Analysis. PLoS One. 2015;10(8):e0134192 10.1371/journal.pone.0134192 . 26244668 PMC4526658

[37] Nicholas MK, Linton SJ, Watson PJ, Main CJ, Decade of the Flags" Working G. Early identification and management of psychological risk factors ("yellow flags") in patients with low back pain: a reappraisal. Phys Ther. 2011;91(5):737–53. 10.2522/ptj.20100224 . 21451099

